# Maternal near miss and predictive ability of potentially life-threatening conditions at selected maternity hospitals in Latin America

**DOI:** 10.1186/s12978-016-0250-9

**Published:** 2016-11-04

**Authors:** Bremen De Mucio, Edgardo Abalos, Cristina Cuesta, Guillermo Carroli, Suzanne Serruya, Daniel Giordano, Gerardo Martinez, Claudio G. Sosa, João Paulo Souza, the Latin American Near Miss Group (LANe-MG), Luis Mainero, Ricardo Fescina, Rafael Aquino, Edgard Iván Ortiz, María Florencia Valacco, Yanina Buono, Daniel Nowacki, Amparo Ramírez, Javier Fonseca, Laura Margarita Bello Alvarez, Cesar Enrique Amores Espin, William E. Arriaga, Rigoberto Castro, Wendy Cárcamo, Liliana Valdez Gil, Miriam Ovando Aldana, Roberto Montenegro, Rodrigo B. Velarde, Rubén Ruttia, Fátima Ocampos, Segundo Acho Mego, Paulino Díaz Ozoria, Gerardo Vitureira, Mario Pérez

**Affiliations:** 1 Latin American Center for Perinatology/Panamerican Health Organization, Montevideo-Uruguay, Av. Brasil 2697, 1er piso, Montevideo, 11600 Uruguay; 2 Rosarino Center for Perinatal Studies, Moreno 878, 6to piso, Rosario, S2000DKR Santa Fe Argentina; 3 Department of Social Medicine Ribeirão Preto Medical School, University of São Paulo, Avenida dos Bandeirantes, 3900, Ribeirão Preto, 14900-000 São Paulo Brazil

**Keywords:** Maternal Near Miss, Potentially Life-Threatening Conditions, Severe Maternal Outcomes, Maternal Mortality, Obstetric Complications, Pregnancy and Childbirth

## Abstract

**Background:**

Every year millions of women around the world suffer from pregnancy, childbirth and postpartum complications. Women who survive the most serious clinical conditions are regarded as to have experienced a severe acute maternal complication called *maternal near miss* (MNM). Information about MNM cases may complement the data collected through the analysis of maternal death, and was proposed as a helpful tool to identify strengths and weaknesses of health systems in relation to maternal health care. The purpose of this study is to evaluate the performance of a systematized form to detect severe maternal outcomes (SMO) in 20 selected maternity hospitals from Latin America (LAC).

**Methods:**

Cross-sectional study. Data were obtained from analysis of hospital records for all women giving birth and all women who had a SMO in the selected hospitals. Univariate and multivariate adjusted logistic regression models were used to assess the predictive ability of different conditions to identify SMO cases. In parallel, external auditors were hired for reviewing and reporting the total number of discharges during the study period, in order to verify whether health professionals at health facilities identified all MNM and Potentially life-threatening condition (PLTC) cases.

**Results:**

Twenty hospitals from twelve LAC were initially included in the study and based on the level of coverage, 11 hospitals with a total of 3,196records were included for the final analysis. The incidence of SMO and MNM outcomes was 12.9 and 12.3 per 1,000 live births, respectively. The ratio of MNM to maternal death was 19 to 1, with a mortality index of 5.1 %. Both univariate and multivariate analysis showed a good performance for a number of clinical and laboratory conditions to predict a severe maternal outcome, however, their clinical relevance remains to be confirmed. Coherence between health professionals and external auditors to identify SMO was high (around 100 %).

**Conclusions:**

The form tested, was well accepted by health professionals and was capable of identifying 100 % of MNM cases and more than 99 % of PLTC variables. Altered state of consciousness, oliguria, placenta accrete, pulmonary edema, and admission to Intensive Care Unit have a high (LR+ ≥80) capacity to anticipate a SMO.

**Electronic supplementary material:**

The online version of this article (doi:10.1186/s12978-016-0250-9) contains supplementary material, which is available to authorized users.

## Plain English summary

The objective of this study is to evaluate the performance of a systematized form to detect severe maternal outcomes in selected maternity hospitals from Latin America.

Data were obtained from analysis of hospital records for all women giving birth and all women who had a severe maternal outcome (SMO) in selected hospitals. A cross sectional study was designed to evaluate and validate the new systematized form and the performance to identify maternal near miss (MNM) and Potentially life-threatening conditions (PLTC) cases. The incidence of SMO and MNM outcomes was 12.9 and 12.3 per 1,000 live births, respectively. The ratio of MNM to maternal death was 19 to 1, with a mortality index of 5.1 %. The use of a systematized form showed a good performance for a number of clinical and laboratory conditions to predict a severe maternal outcome. The form tested, was well accepted by health professionals and was capable of identifying 100 % of MNM cases and more than 99 % of PLTC variables. Altered state of consciousness, oliguria, placenta accrete, pulmonary edema, and admission to Intensive Care Unit have a high capacity to anticipate a SMO.

## Background

In 2015, the World Health Organization (WHO) estimated more than 300,000 maternal deaths worldwide [1]; and around ten million women suffered from complications related to pregnancy, childbirth and postpartum worldwide [2–8]. It is considered that women, who survive the most serious clinical conditions have suffered a severe acute maternal complication called maternal near miss (MNM) [9–11]. Comprehensive studies concluded that information on MNM is helpful to identify health systems’ strengths and weaknesses related to maternal health care. MNM cases have many characteristics in common with maternal deaths and could become a direct source of information on the hurdles that women have to overcome following an acute complication. Such cases may complement the information collected through maternal death analysis [11–15]. Lack of reliable information to assess this problem is a critical issue, whose gap has been partially filled with information derived from two multicenter cross sectional studies, one from Brazil [16] and the other conducted by the WHO in 29 countries [17]. By the same token, the Pan American Health Organization (PAHO) proposed the development of a systematized form to routinely collect and analyze data on maternal health and quality of health care. This form particularly focuses on severe maternal morbidity and mortality so Latin American health facilities are more aware of the he current situation of MNM cases.

The Perinatal Information System consists of basic perinatal clinical records (PCR) with complementary forms and charts and a software package for personal computers that was developed by the Latin American Center for Perinatology (CLAP) from WHO/PAHO and is currently used by 28 countries from the Americas. A standardized form and an application tool was added to use as a data collection form for MNM. According to WHO, a maternal near-miss case (MNM) is defined as “a woman who nearly died but survived a complication that occurred during pregnancy, childbirth or within 42 days of termination of pregnancy” and “potentially life-threatening conditions” (PLTC) is an extensive category of clinical conditions, including diseases that can threaten a woman’s life during pregnancy and labor and after termination of pregnancy. In practical terms, women are considered near miss cases when they survive life-threatening conditions (i.e. organ dysfunction). Additionally, severe maternal outcomes (SMO) are maternal near miss cases and maternal deaths [18].

Therefore, the main purpose of this study was to evaluate the introduction of a standardized form in obstetric routinely practice, in order to collect information on MNM and other Potentially life-threatening conditions (PLTC) variables in LAC selected maternity hospitals. This was done to know the predictive ability of PLTC variables for the identification of severe maternal outcomes (SMO), and to define their acceptance by health professionals (HP).

## Methods

**Fig. 1:**
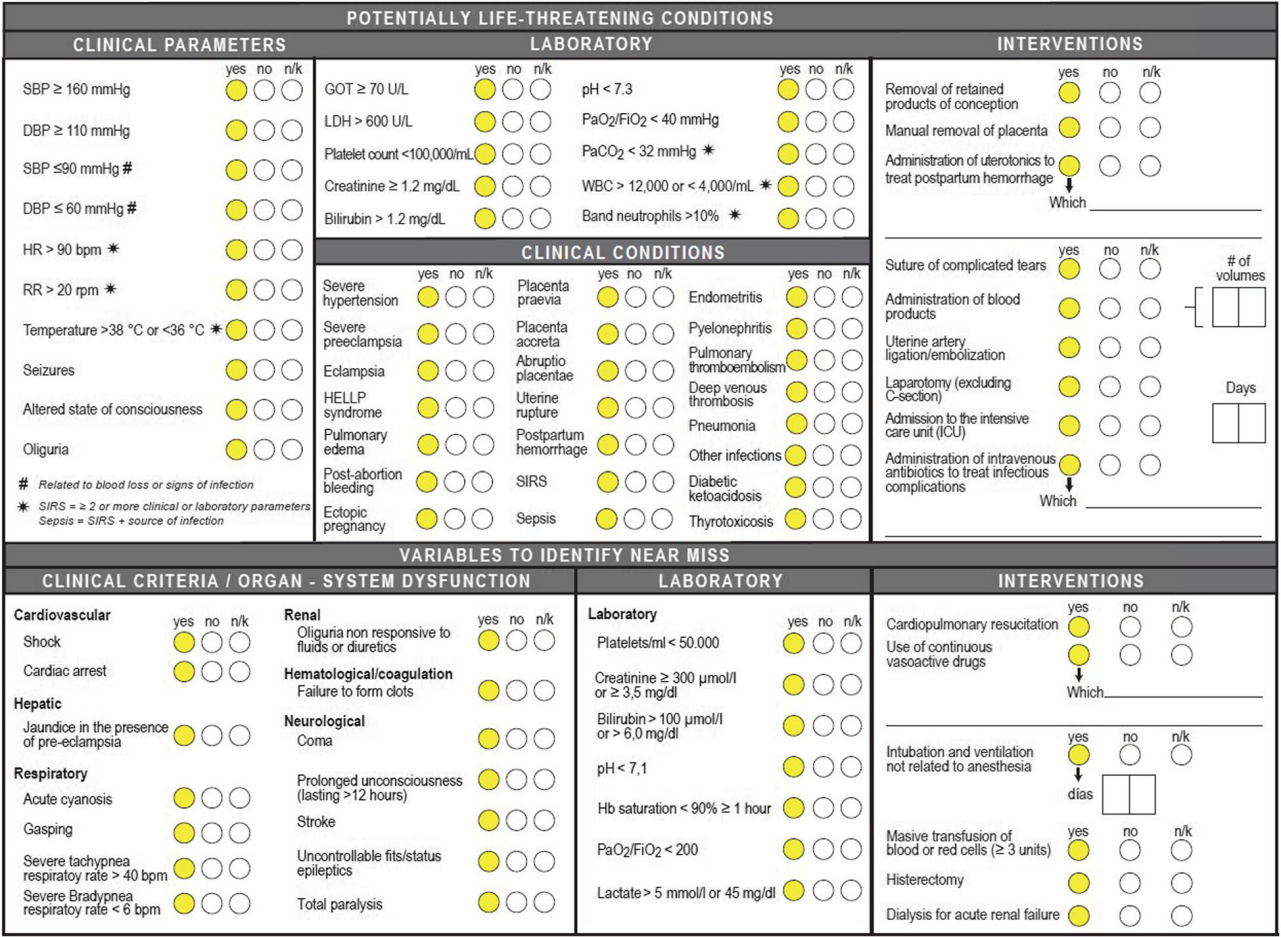
Clinical Perinatal Record (reverse) MNM and PLTC variables

In October 2012, CLAP summoned a group of regional experts to develop an annex form, that would complement the PCR of the Perinatal Information System, based on the definitions and standardized variables included in “The WHO near-miss approach for maternal health” [18]. Once forms were defined and agreed upon (Fig. 1), procedure protocols developed, and manuals of operations written, an invitation was sent through the WHO/PAHO’s country offices to all Spanish-speaking countries in the Americas. Ministries of health of 12 countries accepted to participate in the study, with a total of participating 20 facilities, 19 that met the pre-defined requirement of having 3,000 or more annual deliveries and one facility with 1200 deliveries per year enrolled in the study as well. This process finished in January 2013. In 2013, Nicaragua hosted a training workshop targeted at hospital coordinators responsible for conducting the pilot test at selected facilities and obtaining approvals from local Ethics Institutional Committees to carry out the study. All proposed facilities were accepted, whether they regularly used or did not use the PCR and the CLAP/PAHO’s SIP.

Hospital coordinators trained those professionals who regularly assisted patients at their departments using the training and dissemination modalities that best fitted their context. Fourteen hospitals held workshops, while five organized briefings and/or seminars. When the study ended, hospital coordinators submitted a final standardized report to CLAP, using the “Survey Monkey” survey format. Five external auditors, who were not related with hospital coordinators, were responsible for reviewing and reporting the total number of discharges during the study period. For budgetary reasons, only five external auditors were selected (countries were drawn) to audit health facilities. Taking advantage of the proximity of health facilities in Argentina and Paraguay, the external reviewers hired for those countries, took under their supervision two health facilities in each country. Finally, these auditors supervised seven maternity hospitals (two in Argentina, and Paraguay, one in Colombia, Nicaragua and Uruguay). The objective of the external auditing was to verify whether teams working at health facilities identified PLTC/MNM variables and to confirm which forms were adequately filled in.

The study population consisted of all women giving birth at the participating maternity hospitals during the study period. In addition, the study included all pregnant or postpartum women or women within 42 days of pregnancy termination, regardless of the mode of delivery, and women admitted to the department of obstetrics, surgery, emergency, gynecology, intermediate and/or intensive care units due to any PLTC or MNM condition. It was sufficient to check at least one of the items of the form for MNM or PLTC to consider that as a case (Fig. 1). Before analyzing the predictive ability of each PLTC, a multiple imputation [19] was done to estimate missing data for each PLTC. There were 41 variables with less than 5 % of missing values, five variables with 5–35 % of missing values, and five variables (all corresponding to laboratory values) with more than 35 % of missing values.

The predictive ability of each condition that could potentially identify a case of SMO was assessed. A univariate logistic regression model was used for such assessment, and based on this; positive and negative likelihood ratios (LR) together with their 95 % confidence intervals were calculated. Multivariate analyses were performed separately for the three clinical conditions most significantly associated with MNM and death: hypertensive disorders, postpartum hemorrhage and infections. To this end, a stepwise selection was performed for all variables related to each condition. In all cases, interventions were excluded from the model because it was not possible to establish a time relationship, i.e., whether the intervention took place before or after the occurrence of a SMO. Multivariate logistic models were also adjusted for each of these three conditions in order to estimate the likelihood of a SMO when more than one PLTC occured concurrently. All analyses were conducted using the SAS statistical software (version 9.3).

## Results

The study was planned as a quick 1-month intervention in each maternity hospital; all participating sites completed the study between March and June 2013. Twenty hospitals from twelve countries participated in the study (Additional file 1: Table S1). External auditors identified the same number of MNM cases as those reported by professionals who regularly provided care to women (100 % agreement). For PLTC, there was also a 100 % agreement in six maternity hospitals and a 91.2 % agreement in the remaining facility (total: 99.3 %).

**Fig. 2:**
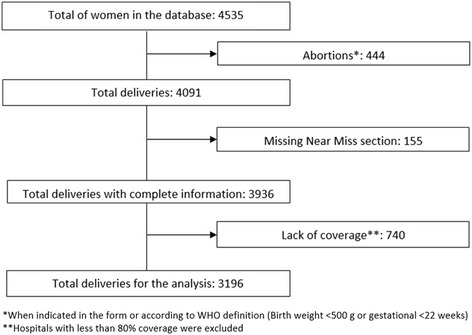
Study flowchart

One hospital from Panama was excluded from analysis, due to the fact that they only entered variables corresponding to the complementary clinical record in the study database. A total of 4,535 women were admitted in the remaining 19 hospitals (deliveries + cases of hospital admissions related to pregnancy or abortion with PLTC/MNM). Four hundred forty-four cases of abortion with SMO were excluded, for the reason that non complicated abortions cases were not always admitted in the participating hospitals, so incidences of abortions with complicatios over the total number of abortions could not be estimated. In addition, 155 cases were excluded because the MNM section had not been completed. The final sample consisted of 3,936 records. In the study period, there were 6,225 births in the participating hospitals, resulting in 63 % coverage. Based on this, it was decided to include in the analysis only those hospitals where coverage was equal to or higher than 80 %, and this resulted in 11 hospitals with 3,196 records (Fig. 2).

In the sample there were two maternal deaths and 37 cases of MNM, resulting in 39 cases of women with a SMO (Table 1). The proportion of mothers aged 10–14 years old or older than 35 years old was higher in the group of women with a SMO than in the overall sample. This is also the case of women with no partners, women without education, nulliparous women, those with only one antenatal care visit or no antenatal care, those with one or more previous cesarean-section (C-section), and those with induced onset of labour or elective C-section or with a delivery that ended in a C-section (Additional file 1: Table S2).

**Table 1:** Maternal Deaths (MD), Maternal Near Miss (MNM), and Severe Maternal Outcomes (SMO) by country

Country	Number of hospitals	Live births	MD	MNM	SMO (MD + MNM)
Argentina	3	762	0	2	2
Colombia	1	334	0	3	3
Dominican Republic	1	133	0	3	3
Ecuador	1	228	0	2	2
Honduras	2	613	1	10	11
Nicaragua	1	477	0	4	4
Paraguay	1	334	1	2	3
Peru	1	315	0	11	11
Total	11	3196	2	37	39

PLTC cases were much more frequent in the group of women with a SMO than in the overall sample, except for neutrophilia with a left shift and SIRS. No differences were observed in relation to pH <7.3, pulmonary thromboembolism, uterine rupture, pneumonia, diabetic ketoacidosis, and Thyrotoxicosis because no cases were recorded (Additional file 1: Table S3). Perinatal outcomes indicate that women with a SMO have a higher rate of preterm births, fetal and neonatal deaths. There is also a higher incidence of newborns with a birth weight of less than 2,500 g and newborns admissions to the Neonatal Intensive Care Unit (Table 2).

**Table 2:** Perinatal results

	All babies n/N (%)	Babies from women with SMO n/N (%)
Preterm birth	414/3113 (13.3)	17/39 (43.6)
Stillbirth	35/3064 (1.1)	5/39 (12.8)
Neonatal death	27/1863 (1.5)	4/18 (22.2)
Admission to SCBU	329/2520 (13.1)	10/25 (40.0)
Birth weight		
<1000 g	18/3053 (0.6)	2/35 (5.7)
1000–1499 g	31/3053 (1.0)	5/35 (14.3)
1500–2499 g	243/3053 (8.0)	5/35 (17.1)
>2500 g	2761/3053 (90.4)	22/35 (68.9)

In relation to overall indicators, the incidence of SMO and MNM was 12.9 and 12.3 per 1,000 live births, respectively. The MNM index per mortality case was 19 to 1, with a mortality index of 5.1 %. Overall, 1 % (32/3,168) of women were admitted to the ICU; 53.1 % of them with a SMO (n = 17). In addition, 46 % of women with a SMO were admitted to the ICU (17/37). The univariate analysis regarding the diagnostic capacity of PLTC for SMOs showed a high prediction of positive results (LR+ ≥20) for the following clinical criteria: altered state of consciousness, oliguria and seizures. Laboratory tests associated to a high LR+ included creatinine ≥1.2 mg/dL; platelet count <100,000 and GOT ≥ 70 U/L. Conditions with a higher predictive efficacy when the test is positive are placenta accreta, pulmonary edema, HELLP syndrome, sepsis and eclampsia. In relation to interventions, the following also have a LR+ ≥20: admission to the ICU, laparotomy and administration of blood products. This group includes certain conditions with a very high predictive and diagnostic capacity when positive (LR+ ≥80): altered state of consciousness, oliguria, placenta accreta, pulmonary edema and admission to the ICU (Additional file 1: Table S3).

An adequate performance (LR+ ≥10–19) was observed in relation to clinical condition indicators, such as postpartum hemorrhage, abruptio placentae, placenta praevia, severe preeclampsia and infections, and in relation to interventions, such as the administration of uterotonics to treat postpartum hemorrhage and the administration of antibiotics to treat infectious complications (Table 3). For the multivariate analysis of hypertensive disorders of pregnancy, variables considered were those included in the form for this condition. Women with altered state of consciousness had almost a five-fold likelihood of developing a SMO: adjusted odds ratio (OR): 4.48; 95 % confidence interval (CI): 2.60–7.74. This is followed by a platelet count lower than 100,000/mL (OR: 3.81, 95 % CI: 2.68–5.43), LDH >600 U/L (OR: 2.64, 95 % CI: 1.66–4.20) and creatinine ≥1.2 mg/dL (OR: 2.28, 95 % CI: 1.35–3.85). The correspondent area under the ROC curve was 0.76.

**Table 3:** Univariate analysis (with data imputation) of PLTC for SMO

	Variable	LR+ (95 % CI)	LR- (95 % CI)	AUC^a^
Clinical parameters	Altered state of consciousness	101.2 (28.2–362.6)	0.9 (0.8–1.0)	0.56
	Oliguria	80.9 (24.4–268.4)	0.9 (0.8–1.0)	0.56
	Seizures	40.5 (7.6–214.5)	0.9 (0.9–1.0)	0.53
	SBP ≥160 mmHg	8.5 (5.3–13.8)	0.7 (0.6–0.9)	0.65
	Temperature >38 °C or <36 °C	7.6 (4.3–13.5)	0.8 (0.6–0.9)	0.61
	SBP ≤90 mmHg	6.9 (3.9–12.3)	0.7 (0.5–1.0)	0.66
	DBP ≥110 mmHg	6.7 (3.5–12.8)	0.8 (0.7–1.0)	0.59
	HR >90 bpm	3.9 (2.9–5.2)	0.5 (0.4–0.7)	0.71
	DBP ≤60 mmHg	3.3 (2.4–4.5)	0.6 (0.4–0.8)	0.68
	RR >20 rpm	2.0 (1.2–3.4)	0.8 (0.7–1.0)	0.57
Laboratory parameters	Creatinine ≥1.2 mg/dL	48.6 (18.6–127.0)	0.8 (0.7–1.0)	0.58
	Platelet count <100 000/mL	35.2 (13.4–92.8)	0.6 (0.4–1.0)	0.67
	AST ≥70 U/L	22.3 (10.3–40.4)	0.8 (0.7–1.0)	0.60
	WBC >12 000 or <4000 /mL	2.2 (1.5–3.2)	0.7 (0.6–0.9)	0.61
	Band neutrophils >10 % (left shift)	1.6 (0.4–6.1)	1.0 (0.9–1.0)	0.51
	LDH >600 U/L	--	--	--
	Bilirubin total > 1.2 mg/dL	--	--	--
	pH <7.3	--	--	--
	PaO2/FiO2 < 400 mmHg	--	--	--
	PaCO2 < 32 mmHg	--	--	--
Conditions	Placenta accrete	161.9 (15.0–1748.7)	0.9 (0.9–1.0)	0.53
	Pulmonary edema	121.4 (20.9–706.6)	0.9 (0.8–1.0)	0.54
	HELLP syndrome	52.0 (23.9–113.0)	0.8 (0.7–0.9)	0.61
	Sepsis	40.5 (12.7–128.8)	0.9 (0.8–1.0)	0.55
	Eclampsia	23.1 (5.0–107.8)	1.0 (0.9–1.0)	0.52
	Postpartum hemorrhage	19.5 (11.6–32.7)	0.7 (0.5–0.8)	0.66
	Abruptio placentae	19.4 (9.8–88.4)	0.9 (0.8–1.0)	0.55
	Other infections	16.8 (8.4–33.3)	0.8 (0.6–1.0)	0.62
	Severe preeclampsia	13.9 (9.1–21.3)	0.6 (0.5–0.8)	0.69
	Placenta praevia	11.0 (3.4–35.4)	0.9 (0.8–1.0)	0.53
	Pyelonephritis	8.6 (0.9–83.3)	1.0 (0.4–2.0)	0.52
	Severe hypertension	6.0 (1.5–24.3)	1.0 (0.9–1.0)	0.52
	Endometritis	5.0 (0.7–37.5)	1.0 (0.9–1.0)	0.51
	SIRS	1.0 (0.5–2.2)	1.0 (0.9–1.1)	0.50
	Uterine rupture	--	--	--
	PTE	--	--	--
	DVT	--	--	--
	Pneumonia	--	--	--
	Diabetic ketoacidosis	--	--	--
	Thyroid storm	--	--	--
Interventions	Admission to the ICU	91.7 (49.4–170.3)	0.6 (0.4–0.7)	0.72
	Laparotomy (excluding C-section)	50.6 (17.3–147.8)	0.9 (0.8–1.0)	0.56
	Administration of blood products	34.0 (3.6–323.0)	0.5 (0.1–2.9)	0.74
	Uterotonics to treat hemorrhages	15.0 (9.5–23.5)	0.6 (0.5–0.8)	0.68
	Use of IV antibiotics to treat infectious complications	14.4 (6.1–34.1)	0.5 (0.3–1.1)	0.72
	Removal of retained products of conception	6.1 (2.6–14.4)	0.9 (0.8–1.0)	0.55
	Manual removal of the placenta	6.1 (1.0–37.4)	0.9 (0.4–2.0)	0.53
	Suture of complicated tears	5.1 (1.3–20.4)	1.0 (0.9–1.0)	0.52
	Uterine artery ligation/embolization	--	--	--

^a^Area under curve

**Fig. 3:**
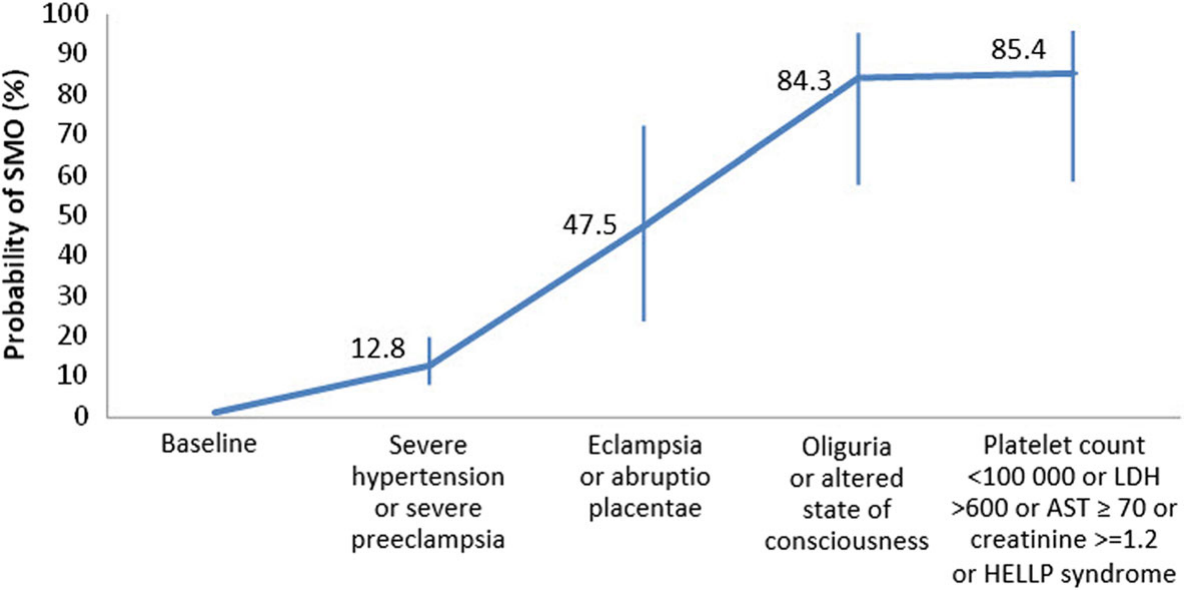
Cumulative estimated probability of SMO in hypertensive disorders of pregnancy

As shown in Fig. 3, the association of more than one of these indicators increases the likelihood of having a SMO. Thus, women with severe hypertension or severe preeclampsia have a 12.8 % likelihood of developing a SMO, and such probability increases to 47.5 % if they also have eclampsia or abruptio placentae. Besides, it increases to 84.3 % if they also have oliguria or altered state of consciousness, and to 85.4 % if, in addition, they have an altered laboratory test.

**Fig. 4:**
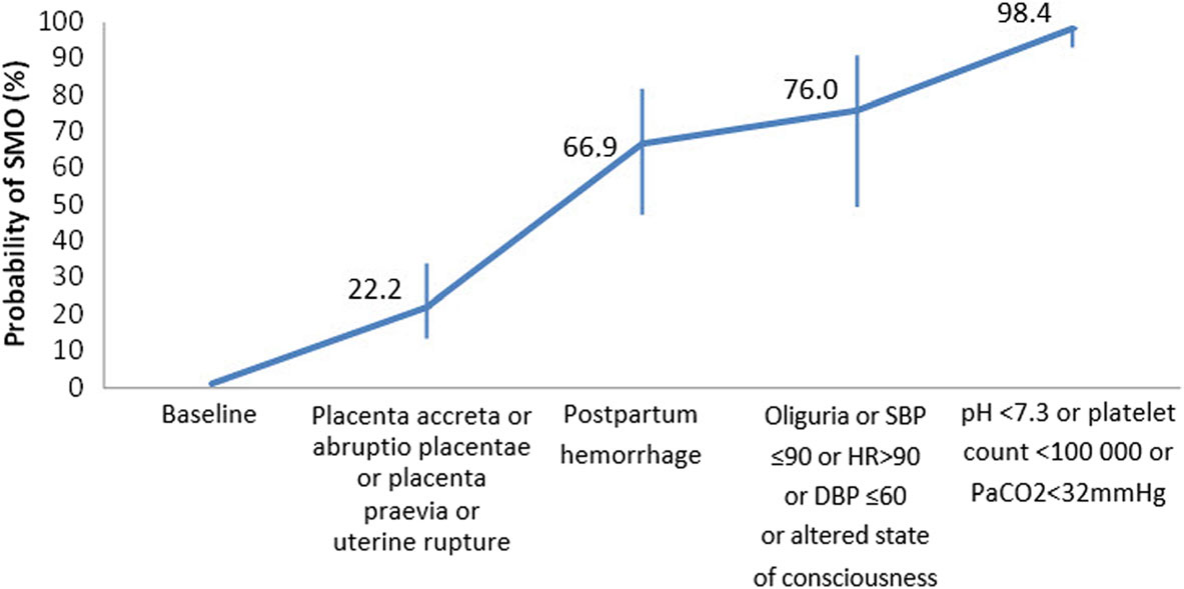
Cumulative estimated probability of SMO in postpartum hemorrhage

For postpartum hemorrhage, a laboratory value of PaCO2 < 32 mmHg indicates a seven-fold increase in the odds of developing a SMO (adjusted OR: 6.99, 95 % CI: 3.89–12.57). This is followed by a platelet count <100,000/mL (adjusted OR: 6.02, 95 % CI: 4.19–8.64), altered state of consciousness (adjusted OR: 4.88, 95 % CI: 2.69–8.83), postpartum hemorrhage (adjusted OR: 2.79, 95 % CI: 1.88–4.12), hypotension (adjusted OR for SBP ≤90 mmHg: 2.29, 95 % CI: 1.60–3.29, and for HR >90 bpm: 1.61, 95 % CI: 1.16–2.21), with an area under the ROC curve of 0.90. When there is an association of these conditions, the likelihood of having a SMO in the presence of a condition is 22.2 %, and it increases to 66.9 % if a woman is diagnosed with postpartum haemorrhage, and to 76 % if she also has oliguria, hypotension or an altered state of consciousness, that increases to 98.4 % if they have any of the laboratory tests with an altered value (Fig. 4).

**Fig. 5:**
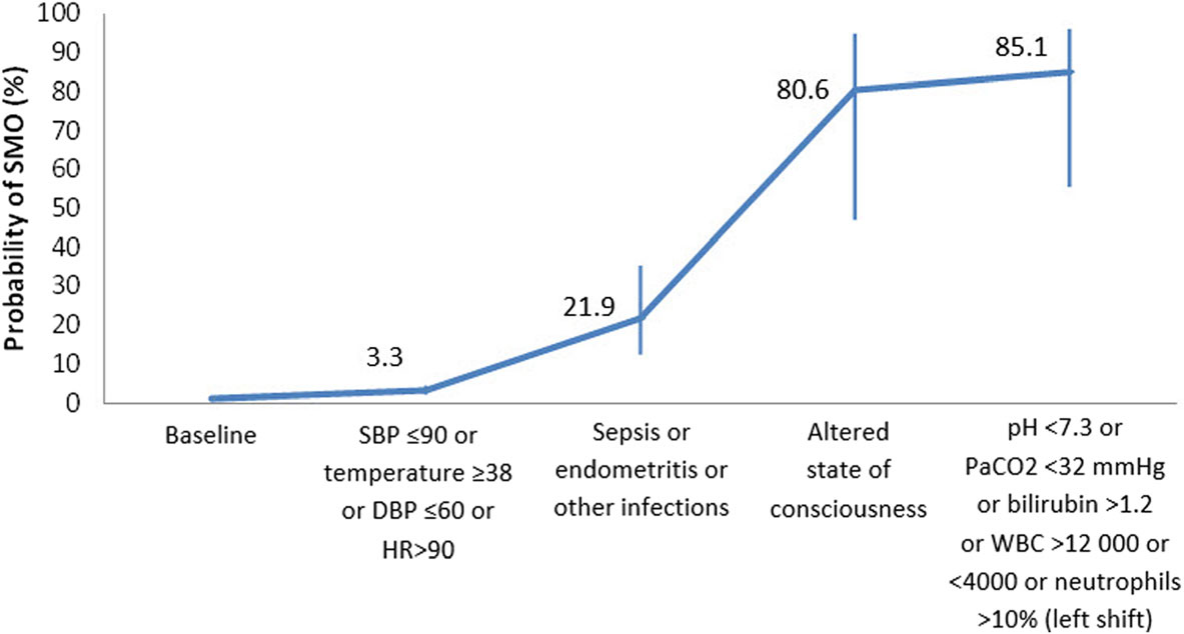
Cumulative estimated probability of SMO in infection

For infections, also a laboratory value of PaCO2 < 32 mmHg indicates a higher than six-fold increase in the odds of developing a SMO (adjusted OR: 6.29, 95 % CI: 3.52–11.24). This is followed by high bilirubin values (adjusted OR: 5.59, 95 % CI: 3.28–9.53), altered state of consciousness (adjusted OR: 4.84, 95 % CI: 2.70–8.66), and pyelonephritis (adjusted OR: 3.29, 95 % CI: 1.67–6.46). Lower, but still significant values were observed in relation to infections, hypotension and tachycardia. The correspondent area under the ROC curve was 0.84. The likelihood of a SMO increases to 3.3 % when there is also hypotension or fever. If these women are also diagnosed with sepsis, endometritis or other infections, such likelihood increases to 21.9 %. When women also have a reduced state of consciousness, their likelihood of having a SMO increases to 80.6 and to 85.1 % if they further have altered laboratory tests (Fig. 5).

## Discussion

The implementation of MNM surveillance at selected hospitals in Latin America is the first step towards the guidance on how to use such tool to improve maternal health. Based on the same guiding principle used for mortality analysis systems, this form is expected to provide health services with systematized information and become a validated tool to monitor potentially life-threatening conditions and identify hurdles that prevent the provision of an adequate and timely care. Such kind of multicenter surveillance was already successfully tried at least twice in Latin America [16] and again proved to be feasible and sensible to identify cases deserving special timely and adequate care.

The form used to identify MNM cases achieved a 100 % case identification in all health facilities. Almost all clinicians participating in the study indicated that identification may be helpful for managers and epidemiologists and for the prevention of potential cases (if correctly audited). There was less agreement on the form’s usefulness for the case clinical management, since parameters show such serious conditions that no further interventions would be required to improve the patient’s diagnosis. In addition, most forms were filled out retrospectively after the case resolution. One potential selection bias during the data collection was the fact that a total of 155 cases were excluded because the MNM section had not been completed. However, in the hospitals that hired external auditors the missing data were for PLTC variables but not for SMO cases. The latter are most of the cases that were admitted to intensive care and the missing data in this patients were scarce,

In relation to the PLTC form, it proved useful to identify almost all cases since in only one health facility the external auditor identified more events than those reported by the attending health care providers. Although it will be necessary to adjust cut-off points for some of the variables, in many cases attending professionals did not comply with the completion requirements indicated in the manual and clinical records themselves. This was particularly the case with the following variables: SBP <90 mmHg, DBP <60 mmHg, HR >90 bpm, RR >20 rpm, WBC >12 000/mL and band neutrophils >10 %.

Some coordinators indicated that the PLTC form was useful because, when completed prospectively, it allowed to warn on the complications at an earlier stage and to take corrective actions. This was particularly the case at the health care facilities where deliveries are attended by professional midwives, nurses and/or general practitioners. However, in most places, the form was completed retrospectively, once the case was closed. At these hospitals, coordinators also stated that the form had not been useful for clinicians dealing with the patient.

Some professionals indicated that certain variables included in the PLTC form are so severe that, when identified, the patient is already managed as MNM. This was especially indicated for certain laboratory tests (pH <7.3, PaO2/FiO2 < 400 mmHg, PaCO2 < 32 mmHg). In addition, these tests had been requested due to the patient’s severe condition, since they are not routinely used. This is confirmed by the fact that more than 35 % of data were missing for five variables (all related to these laboratory tests).

The prevalence of MNM at the 11 maternity hospitals that provided data to the study was 12.9 per 1,000 live births. These values are closer to those reported by the WHO study (13.1 per 1,000 live births) for countries with very high maternal mortality ratios [17] than to those reported for all regions (8.3 per 1,000 live births) or for Latin America alone (8.3 per 1,000 live births data not published).

Univariate analyses on the diagnostic capacity of PLTC for SMO were very high (LR+ ≥80) for clinical conditions such as altered state of consciousness and oliguria, for conditions such as placenta accreta and pulmonary edema, and for interventions such as admission to the ICU. In general, laboratory tests also had an adequate performance when positive (LR+ 20–79). However, these were the PLTC indicators with the highest percentage of missing data (between 5 % for platelet count <100,000/mL and 46 % for PaO2/FiO2 < 400 mmHg); therefore, data imputation was used as a statistical technique for analysis. Given the pragmatic nature of the study, some tests, which are considered to have a high cost or are associated with morbidity (e.g., arterial blood collection) were not requested routinely for all women, only for those who had a severe condition. In addition, all hospitals, except one, retrospectively completed forms, many times upon the patient’s discharge, transfer or death. Some PLTC possibly had more chances of being detected and recorded in women with MNM than in those who did not have MNM, so their diagnostic capacity may have been overestimated. All these reasons call for caution at the time of making conclusions based on these results.

For multivariate analyses, all PLTC in the form that could have been related to the three most common conditions were selected: hypertensive disorders, postpartum hemorrhage and infections. Variables related to clinical interventions in this exploratory model were not included because, given the form characteristics and variable collection modality, it was not possible to establish a time relationship, i.e., whether the intervention occurred before or after the MNM. This is one of the study limitations since some variables (cesarean section, admission to the ICU, laparotomy and administration of blood products) show a high association in the univariate analyses, as reported by other authors [20].

For multivariate exploratory analyses where two or more PLTC occur concurrently, variables were selected in advance based on the logical sequence of their clinical presentation and/or on a diagnosis or management algorithm. Thus, for example, postpartum hemorrhage shows an increased probability of SMO if conditions such as placenta praevia, placenta accreta or early abruptio placentae are diagnosed in advance (during antenatal care visits) or intrapartum; such probability increases in the presence of a confirmed postpartum hemorrhage. If during clinical monitoring, it is confirmed that such woman has reduced urinary output per minute (oliguria) or has clinical signs of arterial hypotension or a reduced state of consciousness, this likelihood increases even more, suggesting the initiation of a hypovolemic shock, which worsens if laboratory tests are also altered. We are aware that other combinations may have also been explored. However, we opted to prioritize the clinical presentation and management criterion for each condition so as to prevent possible spurious associations due to multiple testing, on one side, and to assess the clinical usefulness of the tool, on the other.

Lastly, cases of abortion were excluded from the analysis because not all women with uncomplicated abortions were admitted to participating hospitals. In addition, in some countries, cases of complicated abortions were admitted to reference hospitals which did not participate in the study. Given these reasons, it is not possible to estimate the incidence or contribution of abortion complications in general analyses. This is a major limitation for result generalization because unsafe abortion complications are still one of the leading causes of maternal deaths in the region, and an analysis and better understanding of abortion causes and determinants, and associated morbidities in surviving women, are a priority for public health planners and managers.

## Conclusions

The form tested was well accepted by health professionals. It was capable of identifying 100 % of MNM cases and more than 99 % of PLTC variables. Almost all clinicians participating in the study indicated that it could be helpful for managers and epidemiologists and for the prevention of potential cases (if correctly audited). Some PLTC variables should be reviewed to adjust cut-off points so as to improve its clinical usefulness.

Some professionals indicated that certain variables included in the PLTC form are so severe that, when identified, the patient is already managed as a case of MNM. This was especially indicated for certain laboratory tests (pH <7.3, PaO2/FiO2 < 400 mmHg, PaCO2 < 32 mmHg). In addition, these tests had been requested because of the patient’s severe condition, since they are not routinely used. This is confirmed by the fact that more than 35 % of data were missing for five variables (all related to these laboratory tests). Therefore, some PLTC variables should be reviewed to adjust cut-off points so as to improve its clinical usefulness. Altered state of consciousness, oliguria, placenta accrete, pulmonary edema, and admission to ICU have a high (LR+ ≥80) capacity to anticipate a SMO.

## Additional file

Additional file 1: Table S1. Participating hospitals, recruitment and coverage. **Table S2.** Maternal characteristics. **Table S3.** Potentially life threatening condition distribution among the whole population and women with SMO. (DOC 195 kb)

## Abbreviations

CLAP: Latin American Center for Perinatology
FiO2: Fraction of Inspired Oxygen
HELLP: Hemolysis, Elevated Liver enzymes, Low Platelets
ICU: Intensive Care Unit
LANe-MG: Latin American Near Miss Group
LR: Likelihood Ratio
MNM: Maternal Near Miss
OR: Odds Ratio
Pa02: Partial Pressure of Oxygen
PaCO2: Partial Pressure of Carbon Dioxide
PAHO: Pan American Health Organization
PCR: Perinatal Clinical Record
pH: Potential of Hydrogen
PLTC: Potentially Life-Threatening Conditions
ROC: Receiver Operating Characteristics
SAS: Statistical Analysis System
SIP: Perinatal Information System
SIRS: Systemic Inflammatory Response System
SMO: Severe Maternal Outcomes
WHO: World Health Organization

## References

[1] Trends in maternal mortality: 1990 to 2015. Estimates by WHO, UNICEF, UNFPA, The World Bank and the United Nations Population Division. Geneva: The World Health Organization, 2015. ISBN 978 92 4 156514 1. http://www.who.int/reproductivehealth/publications/monitoring/maternal-mortality-2015/en/.

[2] Lawn JE, Tinker A, Munjanja SP, Cousens S (2006). Where is maternal and child health now?. Lancet.

[3] Filippi V, Ronsmans C, Campbel OMR, Graham W, Mills A, Borghi J (2006). Maternal Health in poor countries: the broader context and a call for action. Lancet.

[4] Khan KS, Wojdyla D, Say L, Gülmezoglu AM, Van Look PF (2006). WHO analysis of causes of maternal death: a systematic review. Lancet.

[5] Ronsmans C, Graham WJ (2006). Lancet Maternal Survival Series steering group. Maternal mortality: who, when, where, and why. Lancet.

[6] Campbell OM, Graham WJ (2006). Lancet Maternal Survival Series steering group. Strategies for reducing maternal mortality: getting on with what works. Lancet.

[7] Paxton A, Maine D, Freedman L, Fry D, Lobis S (2005). The evidence for emergency obstetric care. Int J Gynaecol Obstet.

[8] Costello A, Azad K, Barnett S (2006). An alternative strategy to reduce maternal mortality. Lancet.

[9] Zeeman GG, Wendel GD, Cunningham FG (2003). A blueprint for obstetric critical care. Am J Obstet Gynecol.

[10] Say L, Pattinson RC, Gülmezoglu AM (2004). WHO systematic review of maternal morbidity and mortality: the prevalence of severe acute maternal morbidity (near miss). Reprod Health.

[11] Pattinson RC, Hall M (2003). Near misses: a useful adjunct to maternal death enquiries. Br Med Bull.

[12] Filippi V, Brugha R, Browne E, Gohou V, Bacci A, De Brouwere V (2004). Obstetric audit in resource-poor settings: lessons from a multi-country project auditing ‘near miss’ obstetrical emergencies. Health Policy Plan.

[13] Cochet L, Pattinson RC, Macdonald AP (2003). Severe acute maternal morbidity and maternal death audit--a rapid diagnostic tool for evaluating maternal care. S Afr Med J.

[14] Geller SE, Rosenberg D, Cox SM, Brown ML, Simonson L, Driscoll CA (2004). The continuum of maternal morbidity and mortality: factors associated with severity. Am J Obstet Gynecol.

[15] Say L, Souza JP, Pattinson R (2009). WHO working group on Maternal Mortality and Morbidity classifications. Maternal near miss − towards a standard tool for monitoring quality of maternal health care. Best Pract Res Clin Obstet Gynaecol.

[16] Haddad SM, Cecatti JG, Parpinelli MA, Souza JP, Costa ML, Sousa MH (2011). National Network for the Surveillance of Severe Maternal Morbidity Group. From planning to practice: building the national network for the Surveillance of Severe Maternal Morbidity. BMC Public Health.

[17] Souza JP, Gülmezoglu AM, Vogel J, Carroli G, Lumbiganon P (2013). Moving Beyond Essential Interventions for Reduction of Maternal Mortality (the WHO Multicountry Survey on Maternal and Newborn Health): a Cross-sectional Study. Lancet.

[18] Evaluating the quality of care for severe pregnancy complications: the WHO near-miss approach for maternal health. Geneva: World Health Organization, 2011. WHO Press, World Health Organization, 20 Avenue Appia, 1211 Geneva 27, Switzerland. ISBN 978 92 4 150222 1. http://whqlibdoc.who.int/publications/2011/9789241502221_eng.pdf.

[19] Enders CK. Applied Missing Data Analysis. New York, NY: Guilford; 2010.

[20] Abalos E, Giordano D, Majic C, Morales EM, Peretti JI, Ramos S. Morbilidad severa materna y neonatal: vigilancia en servicios y capacidad de respuesta del sistema de salud. Rev Argent Salud Pública. 2014;5(18):15–23. http://www.rasp.msal.gov.ar/rasp/edicion-completa/RASPVolumen-XVIII.pdf.

