# Supplementary material for: Maternal near miss and predictive ability of potentially life-threatening conditions at selected maternity hospitals in Latin America
Source: Reprod Health. 2016 Nov 4;13:134. doi: 10.1186/s12978-016-0250-9 (PMC5097347; doi:10.1186/s12978-016-0250-9)
Supplement: Additional file 1: Table S1. — Participating hospitals, recruitment and coverage. Table S2. Maternal characteristics. Table S3. Potentially life threatening condition distribution among the whole population and women with SMO. (DOC 195 kb) [file 12978_2016_250_MOESM1_ESM.doc]

**TABLES**

| **Country** | **Hospitals** | **Live births during the study period**  **N** | **Cases in the database**  **N** | **Coverage**  **%** | **Valid coverage**  **%** |
| --- | --- | --- | --- | --- | --- |
| Argentina | M. V. de Martinez | 173 | 164 | 95% | 95% |
|  | Roque Saenz Peña | 163 | 174 | 106%^&^ | 106%^&^ |
|  | Martin | 421 | 424 | 101%^&^ | 101%^&^ |
| Colombia | De Occidente – Kennedy | 313 | 334 | 106%^&^ | 106%^&^ |
|  | Universitario del Valle^1^ | 474 | 108 | 23% |  |
|  | Rafael Calvo | 669 | 48 | 7% |  |
| Dominican Republic | Los Mina | 156 | 133 | 85% | 85% |
| Ecuador | Enrique Garcés | 276 | 228 | 83% | 83% |
| Guatemala | Quetzaltenango^1^ | 448 | 69 | 15% |  |
| Honduras | Roberto Suazo | 231 | 237 | 103%^&^ | 103%^&^ |
|  | ISS Tegucigalpa | 364 | 376 | 104%^&^ | 104%^&^ |
| Mexico | Rafael Pascacio Gamboa^2^ | 687 | 90 | 13% |  |
|  | ISS Estado de México. H. Valle Ceylán^2^ | 377 | 73 | 19% |  |
| Nicaragua | Chinandega | 527 | 477 | 91% | 91% |
| Panama**^*^** | Santo Tomas | 1040 | 1181 | 114% |  |
| Paraguay | Itaugua | 285 | 74 | 26% |  |
|  | San Pablo | 306 | 334 | 109%^&^ | 109%^&^ |
| Peru | Instituto Materno Perinatal | 360 | 315 | 88% | 88% |
| Uruguay | De Clínicas^1^ | 68 | 14 | 21% |  |
|  | Pereira Rossell^2^ | 596 | 312 | 52% |  |
| **Total** | **20** | **7934** | **5165** | **65%** | **97%** |

**Table S1. Participating hospitals, recruitment and coverage**

*Panamá excluded (baseline characteristics, pregnancy, delivery and discharge data missing), **^&^**Includes pregnancy and postpartum admissions, **^1^**Cases with missing MNM or PLTC forms, **^2^**Incorrect ID among forms.

**Table S2**. Maternal characteristics

| **Characteristics** | **All women** | **Women with SMO** |
| --- | --- | --- |
| **Age (years)** | N = 2633 | N = 39 |
|  | n (%) | n (%) |
| 10 – 14 | 26 (1.0) | 1 (2.6) |
| 15 – 19 | 532 (20.2) | 7 (18.0) |
| 20 - 35 | 1836 (69.7) | 25 (64.1) |
| >35 | 239 (9.1) | 6 (15.4) |
|  |  |  |
| **Marital Status** | N = 2415 | N = 38 |
|  | n (%) | n (%) |
| No partner | 346 (14.3) | 9 (23.7) |
| With partner | 2065 (85.5) | 29 (76.3) |
| Other | 4 (0.2) | 0 (0.0) |
| **Education** | N = 2430 | N = 38 |
|  | n (%) | n (%) |
| None | 39 (1.6) | 1 (2.6) |
| Primary | 651 (26.8) | 10 (26.3) |
| Secondary | 1503 (61.9) | 23 (60.5) |
| University | 237 (9.7) | 4 (10.5) |
|  |  |  |
| **ANC** | N = 3103 | N =38 |
| 0 - 1 | 244 (7.9) | 6 (15.8) |
| 2 - 3 | 461 (14.9) | 5 (13.2) |
| 4 or more | 2398 (65.2) | 27 (71.0) |
|  |  |  |
| **Previous births** | N = 3196 | N = 39 |
|  | n (%) | n (%) |
| 0 | 1306 (40.9) | 21 (53.9) |
| 1 - 2 | 1432 (44.8) | 13 (33.3) |
| >2 | 458 (14.3) | 5 (12.8) |
|  |  |  |
| **Previous C-section** | N = 3075 | N = 39 |
|  | n (%) | n (%) |
| 0 | 2538 (82.5) | 30 (76.9) |
| 1 | 390 (12.7) | 6 (15.4) |
| >1 | 147 (4.8) | 3 (7.7) |
|  |  |  |
| **Onset of labour** | N = 3043 | N = 36 |
|  | n (%) | n (%) |
| Spontaneous | 2395 (78.7) | 19 (52.8) |
| Induced | 135 (4.4) | 5 (13.9) |
| C-section | 513 (16.9) | 12 (33.3) |
|  |  |  |
| **Mode of delivery** | N = 3041 | N = 36 |
|  | n (%) | n (%) |
| Vaginal | 1886 (62.0) | 9 (25.0) |
| C-section | 1094 (36.0) | 27 (75.0) |
| Other | 61 (2.0) | 0 (0.0) |

**Table S3**. Potentially life threatening condition distribution among the whole population and women with SMO

|  | **Conditions** | **All women**  **n/N (%)** | **Women with SMO**  **n/N (%)** |
| --- | --- | --- | --- |
| Clinical Parameters | HR >90 bpm | 481/3144 (15.3) | 22/39 (56.4) |
|  | DBP ≤60 mmHg | 509/3171 (16.1) | 20/39 (51.3) |
|  | SBP ≤90 mmHg | 180/3171 (5.7) | 14/38(36.8) |
|  | SBP ≥160 mmHg | 135/3169 (4.3) | 13/39 (33.3) |
|  | RR >20 rpm | 450/3144 (14.3) | 11/39 (28.2) |
|  | Temperature >38 °C or <36 °C | 116/3170 (3.7) | 10/39 (25.6) |
|  | DBP ≥110 mmHg | 105/3174 (3.3) | 8/39 (20.5) |
|  | Altered state of consciousness | 9/3185 (0.3) | 5/39 (12.8) |
|  | Oliguria | 10/3172 (0.3) | 5/39 (12.8) |
|  | Seizures | 6/3186 (0.2) | 2/39 (5.1) |
| Laboratory Parameters | WBC >12 000 or <4000 | 603/2985 (20.2) | 16/38 (42.1) |
|  | Platelet count <100 000 | 46/3037 (1.5) | 14/39 (35.9) |
|  | Bilirubin total >1.2 mg/dL | 19/2152 (0.9) | 11/35 (31.4) |
|  | PaO2/FiO2 <400 mmHg | 6/1723 (0.4) | 6/22 (27.3) |
|  | PaCO2 <32 mmHg | 9/1725 (0.5) | 6/23 (26.1) |
|  | LDH >600 U/L | 20/1907 (1.1) | 5/20 (25.0) |
|  | GOT ≥70 U/L | 37/2073 (1.8) | 8/35 (22.9) |
|  | Creatinine ≥1.2 mg/dL | 16/2279 (0.7) | 6/38 (15.8) |
|  | Band neutrophils >10% (left shift) | 106/2806 (3.8) | 2/36 (5.6) |
|  | pH <7.3 | 0/1732 (0.0) | 0/23 (0.0) |
| Conditions | Severe preeclampsia | 109/3160 (3.5) | 16/38 (42.1) |
|  | Postpartum hemorrhage | 67/3133 (2.1) | 13/35 (37.1) |
|  | Other infections | 57/3119 (1.8) | 10/37 (27.0) |
|  | HELLP syndrome | 23/3160 (0.7) | 9/38 (23.7) |
|  | SIRS | 471/3140 (15.0) | 6/36 (16.7) |
|  | Sepsis | 12/3132 (0.4) | 4/36 (11.1) |
|  | Abruptio placentae | 15/3146 (0.5) | 4/38 (10.5) |
|  | Pulmonary edema | 5/3162 (0.2) | 3/38 (7.9) |
|  | Placenta praevia | 25/3148 (0.8) | 3/38 (7.9) |
|  | Pyelonephritis | 21/3127 (0.7) | 2/37 (5.4) |
|  | Severe hypertension | 29/3154 (0.9) | 2/38 (5.3) |
|  | Eclampsia | 9/3161 (0.3) | 2/38 (5.3) |
|  | Placenta accrete | 3/3143 (0.1) | 2/38 (5.3) |
|  | Endometritis | 17/3123 (0.5) | 1/37 (2.7) |
|  | DVT | 1/3124 (0.0) | 1/37 (2.7) |
|  | PTE | 0/3124 (0.0) | 0/37 (0.0) |
|  | Uterine rupture | 0/3139 (0.0) | 0/37 (0.0) |
|  | Pneumonia | 0/3117 (0.0) | 0/37 (0.0) |
|  | Diabetic ketoacidosis | 0/3121 (0.0) | 0/37 (0.0) |
|  | Thyroid storm | 0/3109 (0.0) | 0/37 (0.0) |
| Interventions | Use of IV antibiotics to treat infectious complications | 120/3161 (3.8) | 18/35 (51.4) |
|  | Administration of blood products | 63/3167 (2.0) | 18/37 (48.7) |
|  | Admission to the ICU | 32/3168 (1.0) | 17/37 (46.0) |
|  | Uterotonics to treat hemorrhages | 95/3144 (3.0) | 14/36 (38.9) |
|  | Laparotomy (excluding C-section) | 13/3169 (0.4) | 5/37 (13.5) |
|  | Removal of retained products of conception | 70/3148 (2.2) | 4/36 (11.1) |
|  | Manual removal of placenta | 45/3158 (1.4) | 3/36 (8.3) |
|  | Suture of complicated tears | 34/3166 (1.1) | 2/36 (5.6) |
|  | Uterine artery ligation/embolization | 2/3167 (0.1) | 2/36 (5.6) |
